# The Opioid/Overdose Crisis as a Dialectics of Pain, Despair, and One-Sided Struggle

**DOI:** 10.3389/fpubh.2020.540423

**Published:** 2020-11-05

**Authors:** Samuel R. Friedman, Noa Krawczyk, David C. Perlman, Pedro Mateu-Gelabert, Danielle C. Ompad, Leah Hamilton, Georgios Nikolopoulos, Honoria Guarino, Magdalena Cerdá

**Affiliations:** ^1^Division of Epidemiology, Department of Population Health, Center for Opioid Epidemiology and Policy, School of Medicine, New York University, New York, NY, United States; ^2^Department of Population Health, School of Medicine, New York University, New York, NY, United States; ^3^Icahn School of Medicine at Mount Sinai, New York, NY, United States; ^4^Mount Sinai Medical Center, Miami Beach, FL, United States; ^5^Graduate School of Public Health & Health Policy, City University of New York, New York, NY, United States; ^6^Department of Epidemiology, College of Global Public Health, New York University, New York, NY, United States; ^7^Center for Drug Use and HIV Research (CDUHR), New York, NY, United States; ^8^Medical School, University of Cyprus, Nicosia, Cyprus

**Keywords:** opioids, overdose, despair, pain, one-sided class war, social conflict

## Abstract

The opioid/overdose crisis in the United States and Canada has claimed hundreds of thousands of lives and has become a major field for research and interventions. It has embroiled pharmaceutical companies in lawsuits and possible bankruptcy filings. Effective interventions and policies toward this and future drug-related outbreaks may be improved by understanding the sociostructural roots of this outbreak. Much of the literature on roots of the opioid/overdose outbreak focuses on (1) the actions of pharmaceutical companies in inappropriately promoting the use of prescription opioids; (2) “deaths of despair” based on the deindustrialization of much of rural and urban Canada and the United States, and on the related marginalization and demoralization of those facing lifetimes of joblessness or precarious employment in poorly paid, often dangerous work; and (3) increase in occupationally-induced pain and injuries in the population. All three of these roots of the crisis—pharmaceutical misconduct and unethical marketing practices, despair based on deindustrialization and increased occupational pain—can be traced back, in part, to what has been called the “one-sided class war” that became prominent in the 1970s, became institutionalized as neo-liberalism in and since the 1980s, and may now be beginning to be challenged. We describe this one-sided class war, and how processes it sparked enabled pharmaceutical corporations in their misconduct, nurtured individualistic ideologies that fed into despair and drug use, weakened institutions that created social support in communities, and reduced barriers against injuries and other occupational pain at workplaces by reducing unionization, weakening surviving unions, and weakening the enforcement of rules about workplace safety and health. We then briefly discuss the implications of this analysis for programs and policies to mitigate or reverse the opioid/overdose outbreak.

## The Opioids/Overdose Crisis as a Dialectics of Pain, Despair and One-Sided Struggle

Millions of words have been written about the opioid/overdose epidemic in the United States, Canada and other countries (1–3). Many of the foremost experts on psychoactive drugs and the treatment of drug problems have written data-filled articles on the topic. So have many social scientists, pundits, and politicians.

This literature makes clear that the opioids/overdose crisis is multifaceted and complex (1, 2, 4). Understanding it takes transdisciplinary knowledge and transdisciplinary theory. In particular, knowledge about chemical dependency and drug treatment is too narrowly focused to come to grips with either the causes of the overdose outbreak or its solutions. Dasgupta, Belesky & Ciccarone provide a useful though general overview of the social and economic roots of the opioid crisis, including its relationships to “deaths of despair” based on changing economic conditions in some communities, and the interactions of these roots with other processes (5, 6). Jalal et al. after careful analysis of the contours of overdose rates in the United States since 1979, framed this as follows (2):

This historical pattern of predictable growth for at least 38 years suggests that the current opioid epidemic may be a more recent manifestation of an ongoing longer-term process. …. Paradoxically, there has been substantial variability with which specific drugs have become dominant in varying populations and geographic locales. ….

Understanding the forces that are holding multiple subepidemics together into a smooth exponential trajectory may be important in revealing the root causes of the epidemic…. Economic and technological “push” factors may be at work to increase supply, such as improved communications and supply chains, efficiencies in drug manufacturing, and expanding drug markets, leading to lower prices and higher drug purities (7, 8). Sociological and psychological “pull” forces may be operative to accelerate demand, such as despair, loss of purpose, and dissolution of communities (9, 10).

Their claim that overdose mortality has been increasing since 1979, that it has been based on a changing variety of drugs, and thus that it is likely the result of social or other processes of a general nature, seems to be accurate (11, 12). A National Academy of Sciences report made a related point (13):

While increased opioid prescribing for chronic pain has been a vector of the opioid epidemic, researchers agree that such structural factors as lack of economic opportunity, poor working conditions, and eroded social capital in depressed communities, accompanied by hopelessness and despair, are root causes of the misuse of opioids and other substances and SUD.

Current efforts to address the opioid/overdose crisis have shown considerable imagination and involve the expenditure of additional funds for treatment of those whose lives have been disrupted by opioid use. Comparatively large amounts of research money are being devoted to this crisis. In particular, the Federal HEALing Communities initiative and other programs for rural communities and for criminal justice populations are devoting considerable money to learn how existing services and their coordination can be improved (14–17). It will be some years before we will know the extent to which these initiatives—which focus on only a small subset of the most impacted communities—actually improve current outcomes. It is important to note, however, that the thrust of these initiatives is to reduce harm to existing opioid users and to help some of them to stop using opioids. These are undoubtedly important goals. They are not the only goal, however. Although programs to reduce opioid prescribing may have some effect, and some community learning about the destruction opioid use can entail is undoubtedly taking place (and may be increased by these initiatives) (5, 18, 19), these programs themselves do not address the social roots of the crisis^1^, and thus are unlikely to reduce the numbers of people beginning to use opioids or other potentially-fatal drugs greatly.

In this article, we first very briefly outline an overall model that ties upstream socioeconomic, political, and community forces to increases in opioid use. We then present an overview of data on the trajectory and magnitude of the epidemics of opioid use and fatal overdose. We then examine some of the proximal roots of this crisis— the role of the pharmaceutical industry and related changes in the funding and regulation of medical care, “communities of despair” (which is a term closely tied in with “deaths of despair') (3, 6), and *pain*, since the use of opioids in many cases is an attempt to alleviate physical and/or psychological pain, with special attention to the roots of such pain in various forms of alienation and in trends in the social nature of work and occupations. We then briefly discuss the implications of this analysis for action.

## A Brief Sociohistorical Model of Upstream Processes and Pathways Which Helped Generate the Opioid/Overdose Epidemic

**Figure 1:**
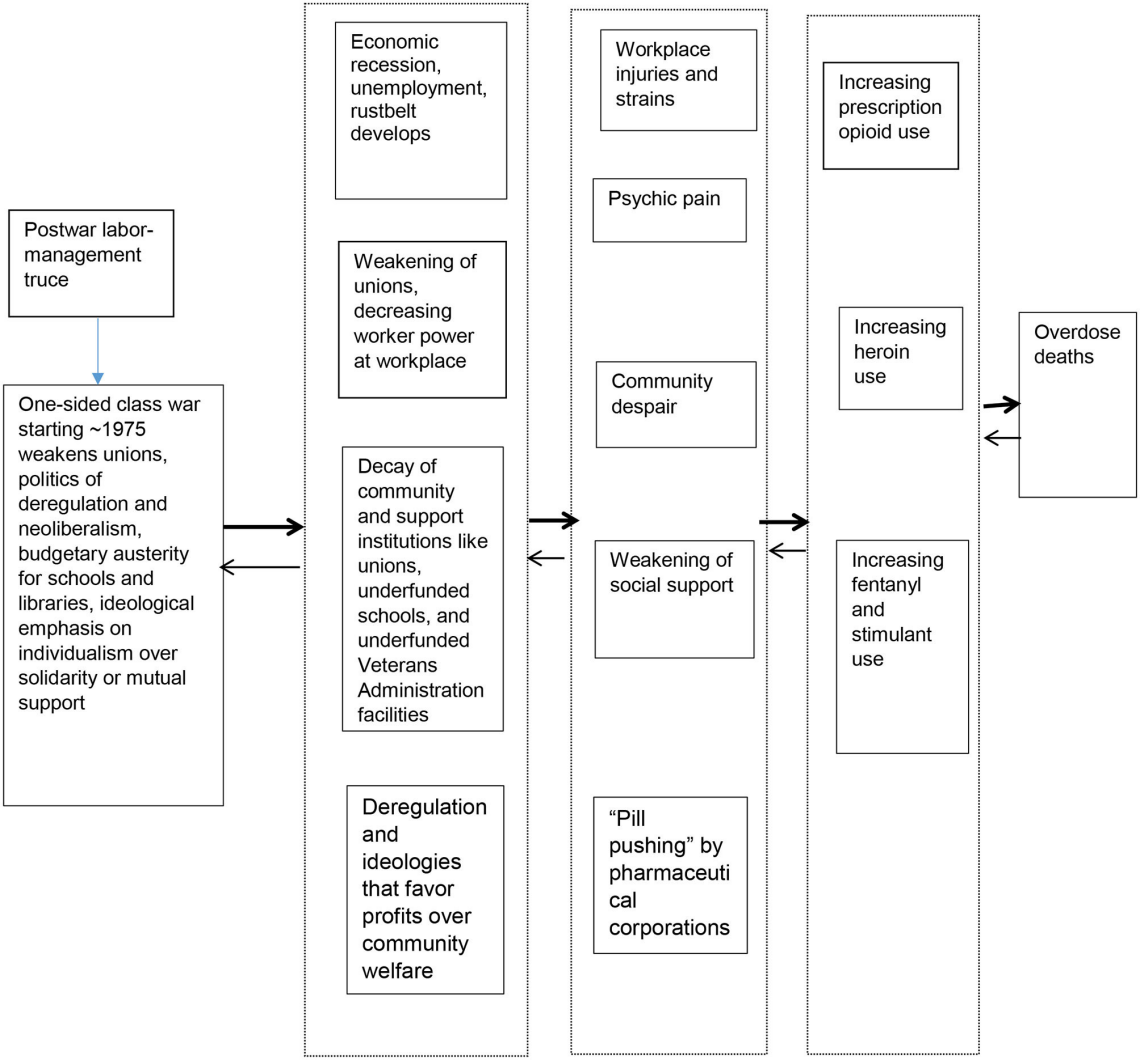
A brief sociohistorical model of upstream processes and pathways through which they helped generate the opioid/overdose epidemic*. *As is discussed in the text, Items on the left seem to contribute causally to items to their right. A degree of reverse causation and of causal influence on items higher or lower in this diagram also seems to take place.

Figure 1 presents an overview of this model. As has been well-documented, the period from 1947 through the early 1970's was one of relative labor-management truce and government focus on economic growth while respecting this peace in almost all industrialized countries (20–25). However, as discussed (and referenced) later in this paper, this truce was replaced by a period of one-sided class war in the 1970s that weakened unions, cut budgets for social services, reduced regulations in transportation (and other) industries in ways that weakened unions, and led to the victory of a political economy of neoliberalism and of ideologies emphasizing individualism and the right of companies to make profits over solidarity or mutual support. This led in the United States to a great growth in economic inequality, to economic recession and the development of the Rustbelt, to weakened unions and reduced ability of workers to defend their working conditions, and to the decay of public schools and other community institutions. This set of events led to communities of despair and to workplace injuries—and thus to physical and psychic pain with reduced community capacity to offer social support to those suffering from these ills. Decreased regulation of pharmaceutical companies and the dominant ideology emphasizing the profitability of companies enabled and perhaps encouraged pharmaceutical companies to introduce new opioid products and to market them aggressively.

This set of processes paved the way for a great increase in prescription opioid use, followed by an increase in the use of heroin, and later of other opioids including fentanyl and of stimulants. Massive increases in overdose mortality were the result.

## Brief Review of the Trajectory and Magnitude of the Epidemics of Opioid Use and of Fatal Overdose

**Figure 2:**
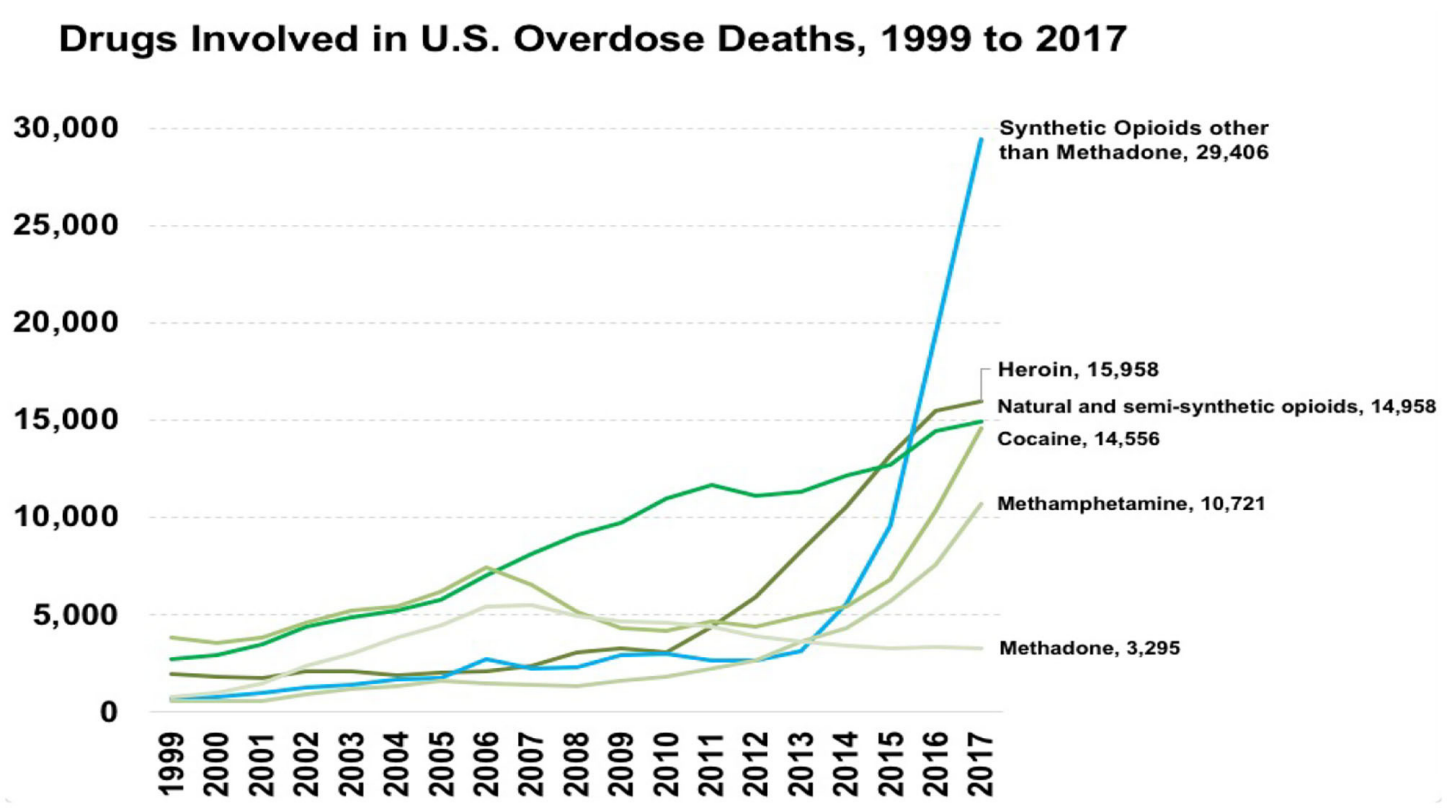
Overdose deaths in the United States, 1999−2017. Source: Centers for Disease Control and Prevention (26)*. *All material in the MMWR series is in the public domain and may be used and reprinted without special permission; citation as to source, however, is appreciated.

The drug overdose epidemic has had multiple phases up to the current time (see Figure 2). While the number of drug overdose deaths has been increasing since 1979 (early in the one-sided class war), it entered a new period in the late 1990s when the first phase of the current epidemic period started with a rapid rise in the use of prescription opioids to treat chronic pain, a subsequent increase in prescription opioid misuse and in prescription opioid overdoses (27). The second phase started in the late 2000s when the prevalence of prescription opioid misuse and overdoses began to stabilize, but heroin use and heroin-related overdoses sharply increased. It has been hypothesized that the rise in heroin use is related to increased trafficking of purer and lower-priced heroin in the illicit market and to increased restrictions placed on the prescription opioid supply (28), with the Great Recession and its socioeconomic effects perhaps serving as a “Big Event” to exacerbate this increase and to produce an increase in methamphetamine use (29–32). A third phase began in 2013, with the introduction of illegally manufactured synthetic fentanyl and related synthetic drugs into the drug market. Overdose deaths spiked, as fentanyl and its analogs are considerably stronger than heroin, and are considerably stronger than heroin, and is often mixed in with other drugs, including other opioids, cocaine, and methamphetamine (33). It has been proposed that we are now in a fourth phase, characterized by polysubstance use, as overdoses involving both opioids and stimulants such as methamphetamine and cocaine have seen an increase, although this may be a continuation of trends in polysubstance use that began after the 2008 economic crisis (29, 34). It is not clear whether the three proximal partial causes of the overall epidemic that we focus on in this paper—pharmaceutical industry activities, community despair, and pain—were differentially important in these three phases, although it is likely that the pharmaceutical corporate contribution was greatest in the first phase.

Although death rates during this period of increased overdose mortality have been highest among American Indians and Non-Hispanic Whites, in recent years overdose mortality rates among African Americans and Hispanics have been increasing more rapidly (35).

## The Role of the Pharmaceutical Industry and Related Changes in the Funding and Regulation of Medical Care

Much current thinking blames the early phases of the current opioid epidemic on the pharmaceutical industry and in some cases on inadequate regulation of this industry, coupled with an increasing push to consider pain as “the fifth vital sign” (36, 37). In 1996, the American Academy of Pain Medicine and the American Pain Society issued a consensus statement on “The Use of Opioids for the Treatment of Chronic Pain,” which argued that opioids should have a role, even a first line role, in the treatment of patients with chronic non-cancer pain (38). Many states then enacted “Intractable Pain Acts” which removed sanctions for prescription of long-term and high-dose opioids. Opioid sales quadrupled between 2000 and 2010. As of 2017, 57 million people (17.4%) in the US received opioid prescriptions, including 15% of men and 20% of women (39).

The pharmaceutical industry played an important role in this push to consider opioids as a safe, non-addictive alternative to no treatment or to the use of other medications without addictive potential for chronic, non-cancer pain. The FDA approved OxyContin in 1996, which Purdue Pharma marketed as non-addictive and effective in treating chronic pain (40). The claim that OxyContin was non-addictive was based on one very flawed and small report (41); this statement is now considered to be factually incorrect (40, 42). The pharmaceutical industry spent tens of millions of dollars annually marketing prescription opioids to physicians, with a subsequent increase in opioid prescribing, including among physicians who received marketing-related payments from the pharmaceutical industry (43). Another important driver of prescription opioid proliferation was the creation of unregulated pain management clinics, or “pill mills,” which functioned as hubs for distribution and sale of prescription opioids across the country (7, 44). For example, in Florida, where such clinics proliferated, and oxycodone-related overdose deaths increased 265% from 2003 to 2009 (8).

## Restriction of the Prescription Opioid Supply and The Rise of the Heroin Market

Federal and state governments responded to growing prescription opioid overdose deaths by regulating prescription opioids (e.g., approving supposedly abuse-deterrent formulations of oxycodone), controlling and monitoring legal access to prescription opioids (e.g., enacting regulations on pain clinics) and shaping prescribing practices (e.g., prescribing guidelines, prescription drug monitoring programs). Prescription opioid overdose deaths stabilized but heroin overdose deaths increased, perhaps because restrictions on the prescription opioid supply led to heroin use among people dependent on opioids (9, 10). (Prescription opioids and heroin have similar pharmacological properties, prescription opioids are often the first opioid used by heroin users, and people with a history of prescription opioid misuse are more likely to begin heroin use than non-users) (28, 45). For example, in one New York City sample of young opioid users, the average time from initiating opioid use to initiating heroin use was <4 years. However, the restricted prescription opioid supply was likely only one contributing factor to the rise in heroin overdose deaths. At the same time as prescription opioid became less available, heroin prices decreased and heroin purity and supply increased (42, 46, 47). After 2013, the introduction of fentanyl into the illegal drug market, and the adulteration of heroin with fentanyl contributed greatly to the rise in heroin overdose deaths.

## Communities of Despair

Another common explanation of the opioid crisis is that it is a reaction to economic and social despair, an argument usually tied to the decline in industrial manufacturing in most of the United States and the “rustbelt communities” it produced. This concept became popular through the works of Case and Deaton (3, 6) which described high death rates among US non-Hispanic whites, particularly among those with cumulative disadvantage and suggested that the prescription of opioids for chronic pain had exacerbated the problem (6).

Case & Deaton's work on this issue were widely publicized. The concepts of deaths of despair and communities of despair were further popularized by an article in *New York Magazine* by Andrew Sullivan (48). Recent evidence that the closing of automobile assembly plants may have increased opioid-related overdose mortality rates in their counties tends to support this argument (49). Relatedly, Pear et al. have shown that non-fatal overdose rates are more common in impoverished localities (50). Thomas et al. reviewed relevant qualitative research studies based in rural areas and found that economic, isolation and other physical conditions, social and policy environments were implicated in opioid-related harm (51).

It should be noted that despair leading to drug use is not a new concept—indeed the heroin and crack epidemics were largely concentrated in impoverished communities of color where lack of jobs, structural racism and over-policing and criminalization created despair in many people (19, 52–55). Opioid use continues to be high in many impoverished minority neighborhoods (56).

Sociological qualitative community studies help explain some of the processes through which changes in economic circumstances lead many people to opioid use or problematic drug or alcohol use (57–59). We will present evidence from two of these conducted in small New England cities. The first, Woonsocket, Rhode Island, is multiracial: In 2017, it was 64% White (60), 18% Hispanic, 6% Black, 7% Asian. The second, Weymouth, Massachusetts, is overwhelmingly (94%) White (61).

Ikeler's study of Woonsocket first provides a historical overview (58). It was a textile center for many years, and was 84% of its workforce was organized by the Congress of Industrial Unions (CIO) in the 1930s. The union established considerable control over workplace life, and over the culture and daily life of the community.

Starting in the 1950s, however, an early Rustbelt experience came to Woonsocket as textile companies moved their production to the US South. A large proportion of Woonsocket's workers, and their descendants, found employment only in short term, precarious work for temp agencies or retail shops. As Ikeler argues:

Attachment to the formal economy or even to a craft or occupation that could provide “ontological security” had declined considerably in post-industrial Woonsocket. …

Yet when work is no longer dependable and its forms increasingly vary—customer service, construction, cab driving, you name it—it ceases to be a dependable site for effort expenditure and identity formation. Precarious workers find alternatives.

These alternatives often involve alcoholism and drug use. As Ikeler goes on to describe:

Alongside fragmented work and absent union experience, subjects described, over and over, the continuity and immanence of substances. Many were not themselves addicts but all witnessed heavy, endemic use in their immediate surroundings.

….Substance use appeared to replace work as the most unifying daily practice; resisting it appeared to replace unionism.

Many of the participants in Ikeler's research both used opioids and other substances and continued to work at those precarious jobs that were available to them. They also fulfilled family roles such as mother and father. In many cases, they did these roles well. Thus, as one participant reported:

“My parents are both junkies,” she told me. “They were good parents though, always emotionally there, just addiction gets annoying.” Corinne had dabbled in opioids herself: “I did heroin only a handful of times and I was like ‘this is stupid’ so I stopped.”But she opined on the reasons for its use around her: “I think it's a hard time,” she said, referring to the economy. “And it's easy—people get depressed, it's easy to grab a bottle or do heroin and just not think for a little awhile. That is why I did it.”

Ikeler goes on to present a complex picture of contradictory tendencies in Woonsocket's community culture. On the one hand, there are forces which lead many people to take up substance use. On the other, neighbors support each other when someone has problems due to drug use and/or when people attempt to quit using drugs.

Ikeler summarizes his analysis as follows:

These stories suggest two things. First, they display the depth and pervasiveness of substance abuse in general and opioid abuse in particular among key groups of contemporary workers. They show this in a way that is not simply parallel to other pursuits, such as work, family, or hobbies, but central and in many ways a replacement.

But second, … they display a reorientation of resistance toward their own habits and those of users around them. Either way, this struggle is internal: internal to the *self* among recovering addicts; internal to *working-class communities* among nonusers.

Class-based resistance …has thus not entirely disappeared in the 21st century. It has in large part been redirected toward substances, the new agents of dependence, rather than employers.

He then briefly discusses the contradictory experiences of West Virginia. He presents data showing that West Virginia had the highest rate of overdose deaths of any state in 2016 and that it had seen the fifth highest decline in union density from 1983-−2016. In spite of this (or perhaps in part as a consequence of this), West Virginia was also the state where the mass teacher strikes of 2018 began, and where they got massive community support, undoubtedly including support from many people who use opioid and their families and neighbors.

Susan Starr Sered conducted an ethnographic sociological study of Weymouth, Massachusetts, a suburban blue collar town south of Boston (57). Her overall analysis complements Ikeler's, in part because she focused less on the experiences of people using drugs and more on issues of what she calls social and cultural capital as described by a wide range of community residents. Like Ikeler, she describes the decline of union employment as leading men (particularly) and women to lose access to long-term full time employment. Unlike Ikeler, and relevant to our discussion of how one-sided class war facilitates both occupational pain and community despair–and thus opioid use, she reports that:

A long-time union member explained, “In working class communities people get injured on the job [and then are] overprescribed pills. If they don't go to work they don't get paid so they fight through injuries. And then one thing leads to the next and the next.”

Although occupational injuries and subsequent use of pain medication made pills accessible, Weymouth residents more often related the current opioid crisis to the “lack of hope for decent [blue collar] jobs,” especially for young men. Thus, several respondents talked about teenagers getting their start with drug mis/use with “finding” pain pills in the medicine cabinets of their blue collar parents. In other words, the parent may have used and perhaps misused prescription pain medication but for the most part in ways that did not significantly interfere with managing a job and daily life. But the kids … took their drug use up to a whole new level.

Like Ikeler, she also shows ways in which the dominance of precarious employment generates a crisis of meaning and of identity. She describes this in terms of “cultural capital; that is, the repertoire of meaningful scripts that help individuals and communities make sense of life's pain, challenges and tedium. Without meaningful scripts, individuals and communities may be more inclined to misuse mind and mood altering substances in order to manage their pain, disappointments and restlessness.” Major sources of decline in such cultural scripts that she identifies include changes in the local school system from one that helped blue collar children form social ties to one oriented to college-based careers focusing on individual material success and problems caused by neoliberal attacks on other public institutions like the Veterans Administration.

She then added:

As access to varied useful and healthy ways to interpret and manage suffering declines or is blocked, opioids and other pain killing and mood changing substances may come to be seen as the only or the most available means of dealing with pain of all kinds.

## Pain: Trends, and its Social Roots

As Sered mentioned, a third proximal cause that has been pointed to for increases in opioid use and overdose deaths is pain, both physical and psychic (57). As discussed above, although pharmaceutical companies increased the supply of opioid pain relievers and engaged in aggressive marketing of these products, initial uptake of these medicines depends, at least in part, on the extent to which people being offered or asking for prescribed opioids, as well as potential prescribers, feel that pain relief would help them. (We specify “initial uptake” because opioid dependence or enjoyment can change the motivations for use).

As we discuss below, there is considerable, though contested, evidence than pain has been increasing in the United States. Supporting such a claim, however, is difficult, because data on pain have many sources of inaccuracy. One potential source of inaccuracy is that to the extent that data depend on self-reported or self-assessed pain levels, there are possibilities both for culturally-induced biases to enter the data, for public attention to pain to increase perceived need for pain relief, and for differential responses by respondents who use different metrics for assessing their own pain levels. In addition, as Dasgupta et al. argue, people sometimes somaticize economic hardship and other stresses into the form of pain, and this could affect both the statistics and the extent of pain suffered by the population (1).

The United States Institute of Medicine considered these issues in a report issued in 2011 (62). It concluded that approximately 100 million Americans suffered from chronic pain. Basing itself in part upon NHANES data, it found that pain had been increasing in the United States. More recently, Nahin et al. used data from the Medical Expenditure Panel Survey to show that non-cancer-related pain that interfered with daily work (including both work outside the home and work in the home) had increased from 1997/98 to 2013/14 among US adults (63). Overall, the proportion of adults reporting painful health condition(s) increased from 32.9% (120 million adults) in 1997/1998 to 41.0% (178 million adults) in 2013/2014. The use of what they classified as *strong* opioids increased more than did non-cancer pain, as did the use of strong opioids within each level of pain interference with work. This trend was particularly strong among those with severe interference due to pain, where the use of strong opioids increased from 11.5% (4.1 million adults) to 24.3% (10.5 million adults) (2).

Nahin et al. also summarize some of the specific causes of pain that have been increasing. These include musculoskeletal conditions, particularly arthritis and spine-related outcomes, and also mental disorders. Keyes et al. point out that both non-medical opioid use and chronic pain and injury are more common in rural areas (64).

### Workplace Sources of Pain

Our model of upstream processes suggests that the one-sided class war leads to less worker control, or even input into, working conditions, safety, and ability to socialize on the job, and thus to loneliness and despair, all of which can lead to more physical and psychic pain (1). The study of what happens at *workplaces* is an issue that many economists, drug researchers, and epidemiologists rarely study (13), even though some earlier reports on increasing opioid deaths dealt with workers' compensation data (65). For example, as shown in the quotation in the Introduction to this paper, the NAS report on *Pain management and the opioid epidemic* mentioned working conditions, but did not substantively examine them. In their otherwise insightful review of the opioid crisis, Dasgupta, Beletsky & Ciccarone do mention working conditions and their association with pain, but do so primarily in connection with poverty and with conditions in poor communities (1). They do not explore the mechanisms or time-trends that might contribute to workplace issues causing *increasing* substance use or overdoses by causing pain. A recent overview show the evidence for and importance of workplace environments in causing physical and psychological pain, opioid use and overdose deaths, but does not tie this into changes in union power and efficacy or to the economic and social changes tied to the one-sided class war (66).

Leukefeld et al. (p. 516) discuss how medicating pain with pain killers had become a part of the culture in Appalachian Kentucky based on the needs of loggers and miners who suffered from occupationally-related pain (67). Specifically, they report that:

Overall, these seventy key informants agreed with the media that the non-medical use and misuse of prescription drugs is widespread and has been a long standing problem with “deep roots” in Appalachian Kentucky and could be part of the “culture.” …This rural drug culture was described by our key informants and others as emerging from loggers who worked with limited power equipment and coal miners who worked bent over in three to four foot high coal mines. The families of loggers and in “coal camp communities” accepted the use of prescription drugs to relieve physical pain and to help wives cope with their depression and their “depressing” surroundings

Buer's *Rx Appalachia* provides additional data about how occupational injuries and Black Lung had led to opioid use, to stressful family situations, and thus to opioid use by family members of those suffering from workplace-induced pain (68, 69).

Cross-sectional data show that people who work in industries and occupations in which workplace injuries or other sources of pain are prevalent are more likely to die of drug-related overdose. *MMWR* reported this for national data for 2007–2012, finding that “Construction occupations had the highest PMRs [proportional mortality ratios] for drug overdose deaths and for both heroin-related and prescription opioid–related overdose deaths. The occupation groups with the highest PMRs from methadone, natural and semisynthetic opioids, and synthetic opioids other than methadone were construction, extraction (e.g., mining, oil and gas extraction), and health care practitioners” (70). A detailed report from Massachusetts for later years (2012−2015) found similarly that construction and extraction occupations were at highest risk; specified that those in health care who were at high risk were health care support staff; and added that those in farming, fishing, and hunting; material moving; installation, maintenance and repair; transportation; production; food preparation and related positions; and building and grounds cleaning and maintenance were also at enhanced risk (71). Cerdá et al. found that, in California, localities with more manual labor industries had a higher rate of hospital discharges for prescription opioid poisoning (72). Most of the industries mentioned above are occupations in which musculoskeletal injuries are frequent. Some of them are also among the occupations in which employment has been growing rapidly; and in these and other industries, the effects of the one-sided class war discussed in depth below also tend to produce more injuries and more physical and psychic pain.

Ompad et al. used National Survey on Drug Use and Health data to compare drug use among construction and extraction workers to that among other workers (73). They found that construction and extraction workers were significantly more likely to report non-prescription opioid use. Missing work due to sickness or injury was also associated with non-prescription opioid use.

## “One-Sided Class War”

We have discussed three major facilitators of the opioids/overdose crisis: actions by pharmaceutical companies, the growth of communities of despair, and increased pain among the population, particularly that owing to injuries, exposures or other sources of pain at work. None of these processes is easy to reverse, although court cases and opioid regulation may have some effect on pharmaceutical industry actions. This suggests it might be useful to investigate whether these three processes have common sources that might be changeable.

Many commentators have investigated these issues. In general, they point to economic globalization, the growth of neoliberal policies and ideologies that include restricting regulation of corporations' activities (including both regulation of pharmaceutical companies and oversight of employers' actions that might lead to injury or harmful exposures of their employees) and result in the movement of much manufacturing and other economic activity away from the Rust Belt. They generally see economic globalization as enforcing a mode of competition that works symbiotically with neoliberalism to create a “race to the bottom” for social welfare and labor protections (24, 25, 74). In some cases, they see these changes as irreversible—which would imply that these drivers of the opioid/overdose crisis might also be irreversible.

The framework we presented at the beginning of this paper and in Figure 1 presents a more hopeful perspective. It frames changes such as globalization, deregulation, and neoliberalism as part of a process of “one-sided class war” through which corporate interests and their political supporters have enforced the dominance of corporate profitability, neoliberal ideology, a global pattern of commodity chains in which production is done where it is cheapest (which forces workers and localities to compete with the poorest countries for employment), and the financial “bottom line” over government policies all over the world. This effort has succeeded in creating declines in unionization, social welfare, protective regulation, and labor standards, and has led to pressures to convert education and health care into profit-making enterprises (75). This one-sided class war framework has been presented in books by Harvey, Davis, and Moody, among others (20–22, 24, 76, 77). The basic thrust of this position is that in the mid- to late-1970s, those who own and run large businesses switched to a more aggressive stance toward unions, social programs, and regulation of business.

One of the first examples of this successful one-sided class war campaign was the “New York fiscal crisis” of 1975 where financial institutions declared that the debts of New York City required massive cutbacks^2^. After New York State established a fiscal control board in charge of the City budget, it made major cuts in municipal services and spending, froze municipal salaries (at a time of rapid increases in the cost of living), laid off large numbers of civil servants, including many union members, raised bus and subway fairs, cut welfare spending, and closed many local hospitals, libraries and fire stations. They also successfully demanded that the unions representing city workers allocate much of their pension funds to the purchase of city bonds—putting the pensions at risk if City bankruptcy took place. As Wallace & Wallace have shown, the closures of fire stations and the general onset of austerity led to massive fires breaking out and spreading in the poorer, mainly Black and Latino, areas of New York (78). These, in turn, led to extreme overcrowding in nearby areas as the dispossessed sought places to live, the decay of school and recreation facilities for youth, and an increase in drug use and vulnerability to infectious disease epidemics such as of tuberculosis and HIV (79).

The one-sided class war took many forms. One of these was the deregulation of the air and trucking industries, which greatly weakened union power and protections for workers in these large industries. More broadly, business increasingly took anti-worker and anti-welfare stands on a wide variety of legislative and administrative issues. This led to some militant rhetoric by some labor union leaders and others—rhetoric which was not by and large backed up by their later actions. A symbolic example of this was a letter made by Doug Fraser, President of the United Automobile Workers, which was at that time a powerful union if and when it chose to strike. This event is described in an article by Jefferson Cowie as follows (75):

In July of 1978, Douglas Fraser … resigned from John Dunlop's Labor-Management Group in a flurry of publicity. The committee had been set up under the Nixon administration to seek out cooperative solutions to labor-management problems and to pass advice along to the White House. Although the group was supposed to reflect the postwar consensus in labor-management relations, Fraser's public resignation and the press conference that accompanied it shredded the fiction of that consensus …. “I believe leaders of the business community, with few exceptions, have chosen to wage a one-sided class war today in this country-a war against working people, the unemployed, the poor, the minorities, the very young and the very old, and even many in the middle class of our society,” he declared. “The leaders of industry, commerce and finance in the United States have broken and discarded the fragile, unwritten compact previously existing during a past period of growth and progress.”

Later, as Cowie describes, the letter argues that:

The new flexing of business muscle can be seen in many other areas. The rise of multinational corporations that know neither patriotism nor morality but only self-interest, has made accountability almost non-existent. At virtually every level, I discern a demand by business for docile government and unrestrained corporate individualism. Where industry once yearned for subservient unions, it now wants no unions at all.

As we discussed above, the increase in overdose deaths began in 1979 and has increased dramatically since then (see Footnote 2). During this time, the dominance of neoliberal ideology and globalization of investment and supply chains proceeded apace, as did the decline in unionized percentages of the workforce. (And as discussed above, Ikeler has presented evidence that the decline of unionization has been a predictor of overdose deaths both longitudinally and cross-sectionally) (58). Elections in both the United States (Reagan) and the United Kingdom (Thatcher) put explicit advocates of neoliberal ideology and policies in charge of two major countries. The Federal Reserve of the United States soon thereafter enacted policies designed to “contract” the economy and thus to increase unemployment—which often meant that companies moved industrial production out of what became the Rust Belt to areas where unions were less prevalent so lower wages could be paid and working conditions worsened in efforts to improve productivity rates.

In both the US and the UK, nationally-coordinated efforts deliberately provoked powerful unions to strike and then mobilized the power of the government and of corporate-owned media to defeat the strikes and (in the US) to de-certify the union (the Professional Air Traffic Controllers Organization). Large-scale decreases in the staffing and the authority of regulatory agencies have also taken place. The power of US unions, and the ability of workers to resist worsening work conditions, has been weakened by bureaucratic internal union regimes; racial/ethnic divisions; political dependence on the Democratic Party; the lingering effects of the red-baiting era and other factors (22, 80–82).

The Great Recession that began in 2007 exacerbated many of these effects by increasing fiscal pressure on governments to implement austerity programs and by increasing unemployment, part-time employment and precarious employment—which have been associated with increased injury rates and other sources of pain—and it should be noted that sharp increases in overdose deaths from opioids and stimulants began shortly thereafter (83).

Pharmaceutical companies were assisted in become massive purveyors of addictive pain medicines by the reduction of government regulatory power over corporations and by the dominance of neoliberal ideologies that support companies' taking actions that yield large profits without regard to “collateral damage”. This was also facilitated by active intervention on the part of pharmaceutical companies to influence clinical pain treatment assessments, guidelines and practices (1). Furthermore, since the era of neoliberalism has been a time in which short-term gains have been emphasized as the key economic indicator, corporate managers and boards of directors were induced to strive for high profits even if some patients might become opioid-dependent as a result. Finally, another thrust of neoliberal thought, and one which has been useful in helping corporations take potentially-profitable activities away from state control, has been the emphasis on *efficiency via cost-cutting* (84). In health care systems, this has taken the form of insurance companies' decisions that doctors and medical organizations can only be reimbursed for performing particular activities, and the pressure this creates for medical institutions to emphasize that doctors process patients rapidly. One aspect of this process was a de-emphasis on behavioral pain therapy and an emphasis on using pharmacotherapy, i.e., analgesics—and particularly opioids—as a way to treat pain (1). In sum, then, the one-sided class war impelled pharmaceutical corporations to maximize their profits lest they go bankrupt or face hostile take-overs, made the production and aggressive marketing of opioid a lucrative way to do this, and reduced regulatory and other counter-pressures that might have deterred them from emphasizing opioids.

The one-sided class war also created communities of despair. Our discussion above showed how it led to the economic abandonment of many communities by manufacturing and other industries that had previously provided stable jobs (with health and other stabilizing benefits); to the destruction or significant weakening of unions that provided social support and identity to many residents; and to the weakening of schools, youth programs, and other community institutions (57, 85, 86). In addition, the neoliberal project that has been a major political form that one-sided class war took has included an ideological emphasis on “individual responsibility” and thus on “individual blame” for failure. Political leaders of both parties, notably including both Presidents Reagan and Clinton, emphasized personal responsibility and the guilt of failure. Thus, neoliberal ideology frames the effects of economic disasters and social institutional decay on each individual and on each family member as being their own fault. This sense of failure, guilt and hopelessness is a major component of communities of despair. Scripts and solidarities that can oppose this sense of guilt and failure were, as both Ikeler and Shered Starr demonstrate, greatly weakened as their institutional bases like unions and some public school systems were attacked by the powerful and as over-policing interacted with these to create a “school-to-prison pipeline” and neighborhood disruption (87). Further, as these same authors also demonstrate, opioids and other psychoactive substances have helped to alleviate (albeit perhaps transiently and with later resultant morbidity) the psychic pain, and drug cultures have created some oases of solidarity.

The discussion above showed that there are positive associations of high-risk occupation and industry with drug use and with fatal overdose. Moody's work, among others, describes pathways through which one-sided class war contributed to increases in both acute and chronic pain. One historic function of unions has been to protect the health and safety of workers. When unions have been stronger, this has been more effective; when they have been weaker, injuries and painful exposures have increased (21, 22, 88–90). Similarly, the ability of individual workers and work groups to defend their safety and health is stronger when their economic security is greater and when solidarity-supporting belief systems are stronger in a community. In the absence of these protections, employers force workers to work more; workers are less likely to hear of and respond to dangerous machinery or risky occupational exposures in time to prevent adverse consequences; and workers are less able to help each other resist management demands that they perform tasks that might lead to musculoskeletal or other injuries. Similarly, many of the employed and some classified as “unemployed” work at part-time or off-the-books jobs with even less than average protection against injury and pain. This is one reason why overdoses are high in agricultural, restaurant and non-union sectors of construction work.

In addition, the lack of worker power on the job often leads to, and perhaps results from, workers' having no time to be sociable or form bonds of solidarity on the job. Further, employers control work-time scheduling so that workers' schedules do not overlap as much as in traditional work. This can contribute to loneliness and to demoralization both at work and in the community—and this, in turn, can open paths to drug use.

Another way in which one-sided class war can lead to higher rates of painful exposures and injuries is through deregulation. Protective government organizations like the Occupational Safety and Health Administration (OSHA) have had their authority to conduct workplace inspections reduced. They have also been starved budgetarily, which has resulted in great reductions in staff availability to conduct inspections. (Similar pressures have also weakened the statistical ability of the government to produce accurate and consistent records of workplace safety and health).

In sum, then, one-sided class war has been a major contributor to the opioid/overdose epidemic by facilitating pharmaceutical companies in their push to increase profits through selling addictive pain medications, specifically opioids; creating communities of despair; and contributing to pain in the population. This suggests that ending (or at least reducing) the one-sided class war might help address the opioid/overdose epidemic.

## International Opioid Epidemics: Evolving Events and Considerations

The discussion and analysis above have focused on the United States, which has been the country most dramatically affected by the opioid epidemic. However, a very similar constellation of forces has led to a significant opioid epidemic in Canada (91–94), affecting every region of the country, albeit unevenly as has occurred in the US. Given that the processes of neoliberalization which contributed to the environment in which the opioid epidemic developed in the US and Canada have also impacted many other countries, reasons for the lack of apparent development of opioid epidemics elsewhere require further study. One potential factor contributing to the varying risk of opioid epidemics in different countries may be the nature of their respective health care systems; health care systems which are primarily for profit and without a single payer, and where high proportions of the population lack health insurance (as in the USA although not Canada) may be at greater risk for a variety of reasons, including that these factors may make if more profitable for pharmaceutical companies to heavily market opioids. Use of restrictive national formularies, which limit the types of opioids which may be used for non-cancer pain, and more restrictive prescribing regulations, may also play a role (95). A study comparing opioid prescribing in eight countries, for example, found that US patients were more likely to receive opioid prescriptions than patients in other countries (96). There are also differences in national and regional regulations which limit various forms of advertising and marketing as well as degrees to which pain treatment guidelines were influenced by pharmaceutical companies. Nonetheless, it is also plausible that opioid and overdose epidemics may occur at different times in different countries due to different balances of “market forces” (meaning neoliberalization, de-industrialization, occupational pain), pharmaceutical marketing efforts and class struggle. Importantly, there are reports that pharmaceutical companies are currently utilizing marketing strategies (such as claims of low addictive potential) that have been curtailed or diminished in the US and Canada, in other countries such as Germany, Italy, Australia, Brazil, Mexico, China and elsewhere (97–99). Also, opioid consumption is increasing in the Netherlands, and the UK NHS has reported that the number of opioid prescriptions has risen dramatically from 2008 to 2018 (100, 101).

There are, of course, many other forces and processes that affect which drugs are available for sale in which locations around a given country or the world. For example, the presence of synthetic fentanyl as a street drug or laced in the heroin drug supply has been reported much more frequently in U.S. relative to most European counties, and likely contributes to differential opioid use and overdose patterns across these regions (102).

Finally, there are clear differences in the extent to which the class war has been one-sided in different countries of the world (22, 77). The United States has long been an outlier among developed countries for lacking a large socialist, social democratic, labor or communist party, for example. In addition, at the start of the period of one-sided class war in the United States, the labor movements in different countries varied widely in political power, organization and capacity to disrupt the economy. The United Kingdom, for example, had a very strong shop stewards movement that was able to organize widespread strikes that drove at least one Prime Minister from power, whereas no comparable capacity existed in the United States. Although the period since then has seen many defeats for workers in Britain, they retain both influence in the Labor Party and capacity for strike action that, at least until 2016, are far greater than comparable forces in the United States. Research is needed on the extent to which these differences underlie international differences in the degrees of regulation imposed on pharmaceutical opioids, the extent of occupationally-induced pain, and/orthe dynamics of communities of despair or the ways in which members of these communities do or do not get involved in opioid use.

More research on the international dimensions and implications of the opioid and overdose epidemics are clearly needed. The discussion in this section suggests that such research will need to consider a wide range of social, economic, political and regulatory factors and will need to consider both the historical records and longitudinal data.

## Implications for Action

Many strategies have been proposed and some implemented for dealing with the opioid/overdose crisis. The Federal and some state governments have expanded drug treatment availability, including evidence-based medications for opioid use disorder. This is much needed; treatment gaps in the United States are huge (103). Efforts are being made to improve coordination among treatment, harm reduction, law enforcement and other community organizations, and to increase availability of naloxone with which to conduct overdose reversals. In addition, the medical community is shifting standards for pain prescribing, and both regulation and law suits have served to reduce pharmaceutical corporations' efforts to increase prescription opioid use. It is too soon to tell whether these efforts will reduce the overdose crisis. It is notable that overdose mortality due to stimulant use has been increasing rapidly in recent years. Furthermore, harm reduction and drug treatment services, as well as drug treatment regulations, budgets, and the cultures of many communities (and of people who use drugs within these communities) have been deeply affected by the COVID-19 pandemic and its related socioeconomic crises, with unknown implications for the future of opioid and stimulant use and of overdose mortality (104–106).

Harm reduction efforts such as expansion of naloxone access, as well as efforts to regulate opioid prescribing through prescription drug monitoring programs and pain management clinic laws, have been associated with reductions in opioid overdose deaths (107–112). Indeed, following investment in multiple efforts to curb high risk prescribing and regulate overall opioid prescribing, prescription opioid overdose rates leveled off (although they did not decrease). At the same time, overdoses involving synthetic opioids continue to increase, as well as overdoses involving both opioids and stimulants.

Furthermore, there is a strong likelihood that in the absence of action to reverse the one-sided class war, conditions in American communities will get worse for a majority of people due to further cuts in services, further rollbacks of safety regulations, and the increasing consequences of climate change and its many ramifications (113, 114). The worsening conditions are likely to increase despair and to produce additional sources of injury and pain. Thus, unless the disruption due to climate change disrupts access to drugs, these changes are likely to induce additional drug use and overdose mortality.

The COVID-19 pandemic and its associated economic crisis may have devastating impacts on efforts to reduce community despair and occupational pain. These events have created enormous costs for state and local governments and have reduced their revenues to a great degree. Political battles over how to make their budgets are likely to occur as long as the economic crisis persists, and to be full of conflict. The one-sided class war has created the conditions under which what Naomi Klein has called the “Shock Doctrine” is likely to be successful (115). The Shock Doctrine describes how corporations and politicians use crises to seize the initiative and cut public services such as schools, welfare and public health and eliminate regulations that limit what corporations can do. These are precisely the types of policies that have facilitated the opioid/overdose crisis. Beyond that, such policies are likely to lead to cuts in treatment for opioid use disorder and other drug treatment approaches, and perhaps weaken harm reduction programs as well.

The analysis in this paper points to counteracting the one-sided class war as a strategy that focuses on an upstream cause of pain, community despair, and pharmaceutical sales of addictive medicines, and that also organizes power to resist Shock Doctrine kinds of attacks. (We would argue that this would have many advantages to many people in addition to those specifically concerned with drug use and overdose, including making it more feasible to reduce greenhouse gas emissions. Those who support or profit from the one-sided class war might disagree).

Counteracting the one-sided class war is not easy, and will involve internal discussion and struggle within the working class among racial/ethnic groups, gender groups and among people with different employment statuses and occupations. These issues have been discussed by scholars and activists from many disciplines (22, 77, 80, 82, 116, 117). We will not propose a fully developed strategy for addressing these issues here. The social and economic disruptions related to COVID-19 have created a rapidly changing sociopolitical and economic environment that poses particular difficulties for strategic planning at this time, although they also offer opportunities for social change (see below).

What we will do is point to two general lines for strategic action. These should be studied and evaluated.

The one-sided class war has been supported politically by the ideologies of neoliberalism that posit individuals and corporations as the building blocks of society and see governments (except in their law enforcement and military mores) as taxing away resources from these building blocks and as limiting individuals' and corporations' freedom to innovate and bring prosperity. One strategy for weakening or reversing the one-sided class war is to attack these ideologies. Indeed, many people have been trying to do this since the 1970s. Articles like this one, which show some of the harmful effects of neoliberal one-sided class war, are indeed inherently part of this strategy.

The other basic strategy is to make the class war two-sided. In recent years, but before COVID-19, mass teacher strikes that had won gains for teachers, other government employees, and school kids had shown that such an approach can make gains. Events during the first half of 2020 have initiated a period of social contestation that seems to create additional avenues for opening up the class war insofar as they have led to mass activism by Black people and their allies around police violence and other issues. It should be remembered that similar movements in the 1960s contributed to increases in both union organizing and active struggle within and by previously organized unions (including struggles over racism within unions) (80, 81, 88). The first half of 2020 has also seen a wave of more wildcat (unofficial) and other strikes than have taken place for decades (118). Mass struggles over how to resolve the COVID-19-related budget deficits in state and local governments are just one form of such likely struggles over the next few years. More generally, efforts to build a mass-based social movement unionism along the lines Moody has put forward, if successful, could do much to make the class war two-sided and to reduce community despair and occupational pain and injury (22).

Community organizing of various sorts can also help blunt and reverse the damages wrought by one-sided class war. Indeed, Moody, Shered Starr and Ikeler all point to ways this can be done. We would add, based on our experience and that of harm reductionists globally, that people who use drugs have set up organizations of their own in some areas that sometimes take part in community and other activist movements. They can be effective members of such community organizing, and can contribute to ensuring that community and union efforts address opioid- and overdose-related workplace and community problems.

In sum, then, one-sided class war has been an important upstream contributor to the chain of causation that has led to, and continues to drive, the opioid and overdose epidemics. Our paper points to important issues for new research to address and to new intervention approaches that might help reduce opioid and overdose problems.

## Author Contributions

SF took overall responsibility for the paper. MC drafted some sections. All authors contributed ideas and contributed to the writing of the paper, and reviewed it and agreed it should be submitted.

## Conflict of Interest

The authors declare that the research was conducted in the absence of any commercial or financial relationships that could be construed as a potential conflict of interest.

## Footnotes

^1^This is not a criticism of these programs. They are important and intelligent programs to determine how best to help people with opioid-related problems. That is a valuable goal—just not the focus of this paper. Likewise, we applaud and support the many grassroots and institutionally-based efforts to embed naloxone availability and use in communities to reduce overdose fatalities.

^2^This is an example of a Structural Adjustment Program being applied to a major city in a core superpower.
